# Supplementary material for: Cost-free lifespan extension via optimization of gene expression in adulthood aligns with the developmental theory of ageing
Source: Proc Biol Sci. 2021 Feb 3;288(1944):20201728. doi: 10.1098/rspb.2020.1728 (PMC7893226; doi:10.1098/rspb.2020.1728)
Supplement: Supplementary Methods, Tables and Figures [file rspb20201728supp1.pdf]

Supplementary materials

Cost-free lifespan extension via optimisation of gene expression in adulthood aligns with the developmental theory of ageing

Martin I. Lind[1∞*], Hanne Carlsson[2*], Elizabeth M. L. Duxbury[2], Edward Ivimey-Cook[2] and Alexei A. Maklakov[2∞]

[1] Animal Ecology, Department of Ecology and Genetics, Uppsala University, Uppsala, SE-75236, Sweden
[2] School of Biological Sciences, University of East Anglia, Norwich NR4 7TJ, UK

* These authors contributed equally to this paper

∞ Corresponding authors
Martin I. Lind: martin.lind@ebc.uu.se
Alexei A. Maklakov: a.maklakov@uea.ac.uk

Methods

Gene expression

Total RNA was extracted from worms homogenised in TRIsure, using an optimised phenol-chloroform protocol, including two chloroform extractions and three ethanol washes of the RNA pellet, to maximise RNA yield and reduce contamination. RNA concentration and purity were checked with Nanodrop spectrophotometry (Thermo Scientific).

Extracted RNA was first DNase treated with 1 unit of RNase-free DNase I (Promega) per 20 µl reaction, following the protocol of the manufacturer, to remove any residual genomic DNA (gDNA) contamination. We then reverse transcribed 68.55 ng of DNase-treated RNA with UltraScript 2.0 reverse transcriptase (RT) and random hexamer primers (both PCR Biosystems). We included a no RT control (NRTC) per RNAi treatment, for which the RT enzyme was substituted for nuclease-free water in the master mix to which the RNA was added. The synthesised cDNA was used undiluted for PCR and qRT-PCR.

To confirm that any contaminating genomic DNA had been removed, we performed a standard PCR with a 10 µl reaction and an annealing temperature of 60°C. We ran 5 µl of the PCR reaction on a 1% agarose gel using ethidium bromide and confirmed both the absence of amplification in the NRTCs verifying the successful removal of any contaminating gDNA, and also the successful amplification of cDNA for all primer pairs.

The qRT-PCR was performed on an Applied Biosystems 7500 Real-Time PCR System using a 2X qPCRBIO Lo ROX Sybr Green kit (PCR Biosystems) with the following PCR cycle: 95°C for two minutes followed by 40 cycles of: 95°C for 5 s followed by 60°C for 30 s. The total reaction volume was 20 µl. We used primers specific for the target gene of interest and for a reference gene- the housekeeping gene, *actin-3* (T04C12.4), commonly used for *C. elegans* (Weick et al., 2014; Akay et al., 2017; Senchuk et al. 2018). Primer sequences are listed in Table S19. Primers were designed based on MIQUE guidelines (Bustin et al., 2009) and taken from (Chauve et al., 2020) for the target genes of interest and (Akay et al., 2017) for *actin-3.*

Two qRT-qPCR reactions (technical replicates) were carried out per sample with both the target gene of interest and the reference gene primers, to check for repeatability. Samples were spread across two plates with all the samples for three genes of interest on the first plate and for the remaining two genes (complete with the corresponding untreated control samples) on the second plate, to control for any minimal plate effects. We also included two negative template controls (nuclease-free water substituted for cDNA) and one NRTC per primer pair per plate, to test for any contamination.

Table S1. The number of individuals of each gene and treatment combination that were excluded from the *lifespan analysis* because of a bacterial infection on the NGM plate. The numbers are a multiply of 10, since the whole plate was always removed.

Gene	Control	Lifelong	Adulthood	Post-reproductive
age-1	10	0	0	0
raga-1	10	0	0	0
nuo-6	0	10	0	0
ifg-1	10	0	0	0
ife-2	10	10	20	10

Table S2. The number of individuals of each gene and treatment combination that were excluded from the *reproduction analysis* because of a bacterial infection on the NGM plate.

Gene	Control	Lifelong	Adulthood
age-1	4	0	0
raga-1	5	2	2
nuo-6	0	0	0
ifg-1	0	0	0
ife-2	5	2	7

Table S3. Lifespan. The effect of age-specific down-regulation of each gene on lifespan. Treatment contrast from Cox proportional hazard models, presented for each gene.

Gene	Treatment contrast	coef.	SE	z	p
age-1	Lifelong	-1.76	0.191	-9.19	**<0.001**
	Adulthood	-1.11	0.329	-6.30	**<0.001**
	Post-reproductive	-0.66	0.517	-3.89	**<0.001**
raga-1	Lifelong	0.135	0.191	0.71	0.480
	Adulthood	-0.647	0.202	-3.21	**0.001**
	Post-reproductive	-0.454	0.193	-2.34	**0.019**
nuo-6	Lifelong	-1.52	0.218	-5.49	**<0.001**
	Adulthood	-0.92	0.263	-3.51	**<0.001**
	Post-reproductive	-0.06	0.258	-0.23	0.820
ifg-1	Lifelong	0.07	0.17	0.43	0.670
	Adulthood	-0.05	0.18	-0.29	0.770
	Post-reproductive	-1.16	0.20	-5.86	**<0.001**
ife-2	Lifelong	-1.52	0.17	-8.89	**<0.001**
	Adulthood	-0.69	0.50	-4.59	**<0.001**
	Post-reproductive	-0.39	0.68	-2.61	**0.009**

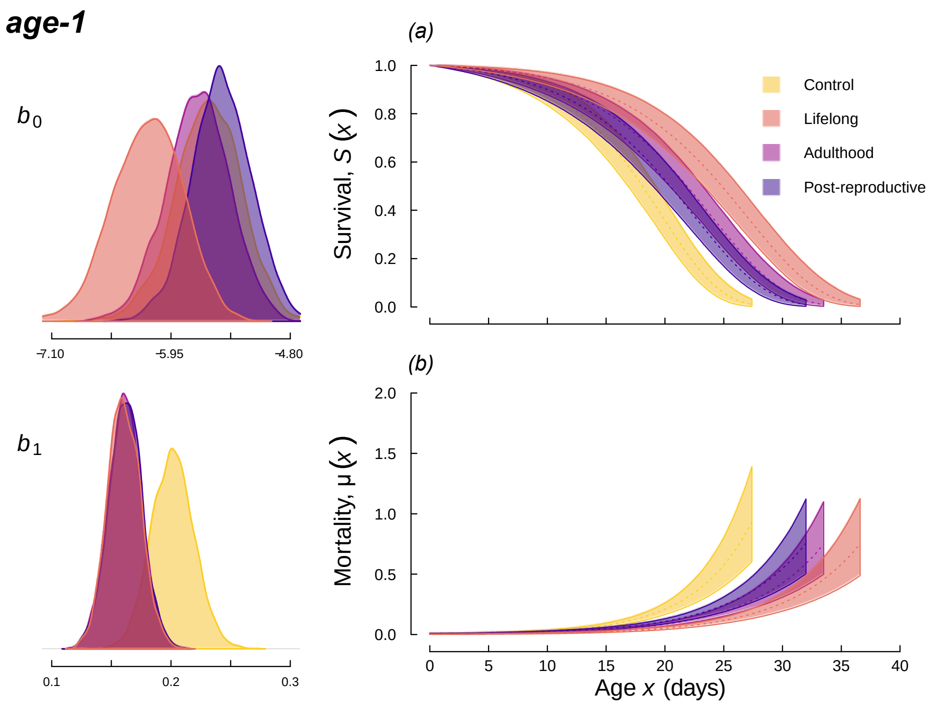

Figure S1. Mortality *age-1*. Survival (a) and mortality (b) curves with 95% confidence interval for each treatment, fitted for *age-1* using a Gompertz model with simple shape in *BaSTA*. The left column corresponds to the two mortality parameters; see the method section for a detailed description.

Table S4. Mortality *age-1*. Coefficients for the mortality parameters estimated for *age-1* using a Gompertz model with a simple shape.

Coefficient	Treatment	Estimate	SE
b_0	Control	-5.592	0.320
	Adulthood	-5.693	0.306
	Post-reproductive	-5.464	0.287
	Lifelong	-6.174	0.337
b_1	Control	0.201	0.018
	Adulthood	0.161	0.014
	Post-reproductive	0.162	0.014
	Lifelong	0.161	0.014

Table S5. Mortality *age-1*. KLDC values for each pairwise comparison of every parameter of the mortality rate model for *age-1*. Values above 0.8 are considered substantially different and are indicated in bold. See method for details.

Comparison	b_0	b_1
Adulthood - Control	0.526	**0.978**
Post-reproductive - Control	0.548	**0.976**
Post-reproductive - Adulthood	0.630	0.501
Lifelong - Control	**0.895**	**0.980**
Lifelong - Adulthood	0.838	0.501
Lifelong - Post-reproductive	**0.962**	0.502

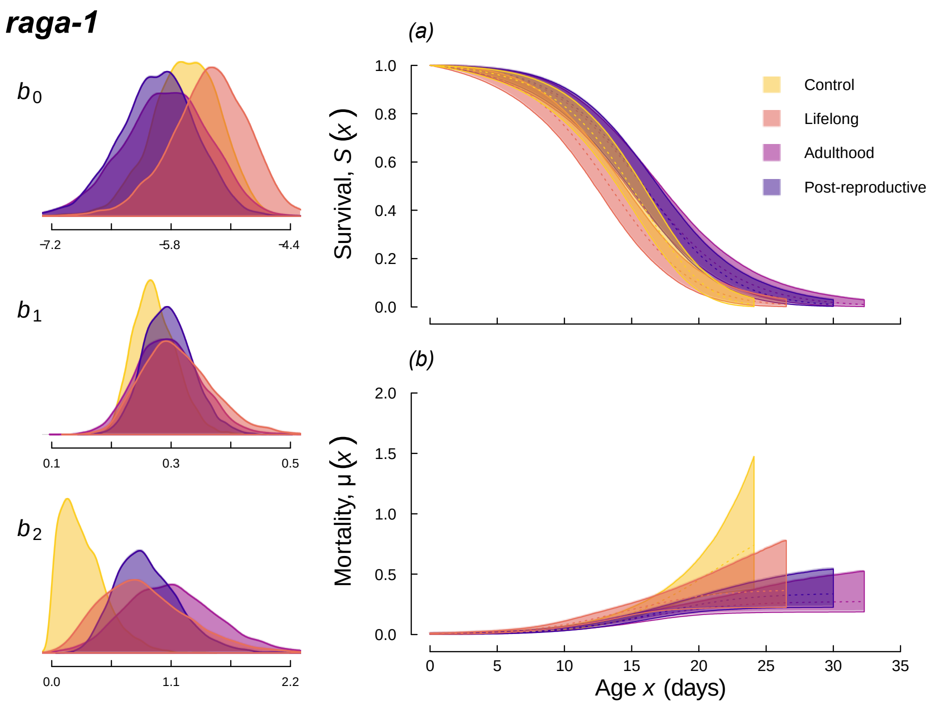

Figure S2. Mortality *raga-1*. Survival (a) and mortality (b) curves with 95% confidence interval for each treatment, fitted for *raga-1* using a logistic model with simple shape in *BaSTA*. The left column corresponds to the three mortality parameters; see the method section for a detailed description.

Table S6. Mortality *raga-1*. Coefficients for the mortality parameters estimated for *raga-1* using a logistic model with a simple shape.

Coefficient	Treatment	Estimate	SE
b_0	Control	-5.628	0.396
	Adulthood	-5.865	0.505
	Post-reproductive	-5.961	0.447
	Lifelong	-5.352	0.450
b_1	Control	0.273	0.036
	Adulthood	0.296	0.054
	Post-reproductive	0.296	0.041
	Lifelong	0.310	0.059
b_2	Control	0.289	0.209
	Adulthood	1.103	0.417
	Post-reproductive	0.885	0.295
	Lifelong	0.867	0.414

Table S7. Mortality *raga-1*. KLDC values for each pairwise comparison of every parameter of the mortality rate model for *raga-1*. Values above 0.8 are considered substantially different and are indicated in bold. See method for details.

Comparison	b_0	b_1	b_2
Adulthood - Control	0.591	0.635	**0.974**
Post-reproductive - Control	0.639	0.587	**0.964**
Post-reproductive - Adulthood	0.517	0.535	0.636
Lifelong - Control	0.603	0.727	**0.932**
Lifelong - Adulthood	0.723	0.520	0.570
Lifelong - Post-reproductive	**0.800**	0.583	0.545

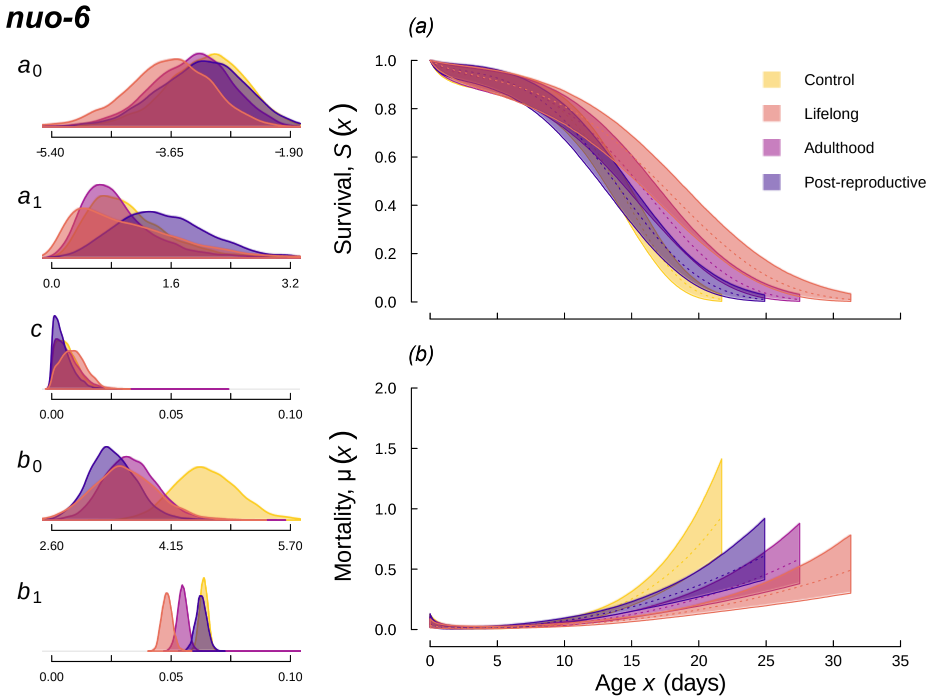

Figure S3. Mortality *nuo-6*. Survival (a) and mortality (b) curves with 95% confidence interval for each treatment, fitted for *nuo-6* using a Weibull model with bathtub shape in *BaSTA*. The left column corresponds to the five mortality parameters; see the method section for a detailed description.

Table S8. Mortality *nuo-6*. Coefficients for the mortality parameters estimated for *nuo-6* using a Weibull model with a bathtub shape.

Coefficient	Treatment	Estimate	SE
a_0	Control	-3.148	0.582
	Adulthood	-3.368	0.584
	Post-reproductive	-3.213	0.628
	Lifelong	-3.692	0.630
a_1	Control	1.030	0.531
	Adulthood	0.890	0.491
	Post-reproductive	1.495	0.624
	Lifelong	1.024	0.704
c	Control	0.006	0.004
	Adulthood	0.007	0.005
	Post-reproductive	0.005	0.004
	Lifelong	0.009	0.005
b_0	Control	4.602	0.411
	Adulthood	3.625	0.344
	Post-reproductive	3.394	0.310
	Lifelong	3.549	0.416
b_1	Control	0.064	0.002
	Adulthood	0.055	0.007
	Post-reproductive	0.063	0.002
	Lifelong	0.048	0.002

Table S9. Mortality *nuo-6*. KLDC values for each pairwise comparison of every parameter of the mortality rate model for *nuo-6*. Values above 0.8 are considered substantially different and are indicated in bold. See method for details.

Comparison	a_0	a_1	c	b_0	b_1
Adulthood - Control	0.534	0.521	0.506	**0.981**	**0.957**
Post-reproductive - Control	0.506	0.647	0.540	**0.997**	0.601
Post-reproductive - Adulthood	0.518	0.740	0.564	0.614	**0.932**
Lifelong - Control	0.667	0.525	0.586	**0.980**	**1.000**
Lifelong - Adulthood	0.568	0.567	0.553	0.527	**0.922**
Lifelong - Post-reproductive	0.625	0.592	0.705	0.583	**1.000**

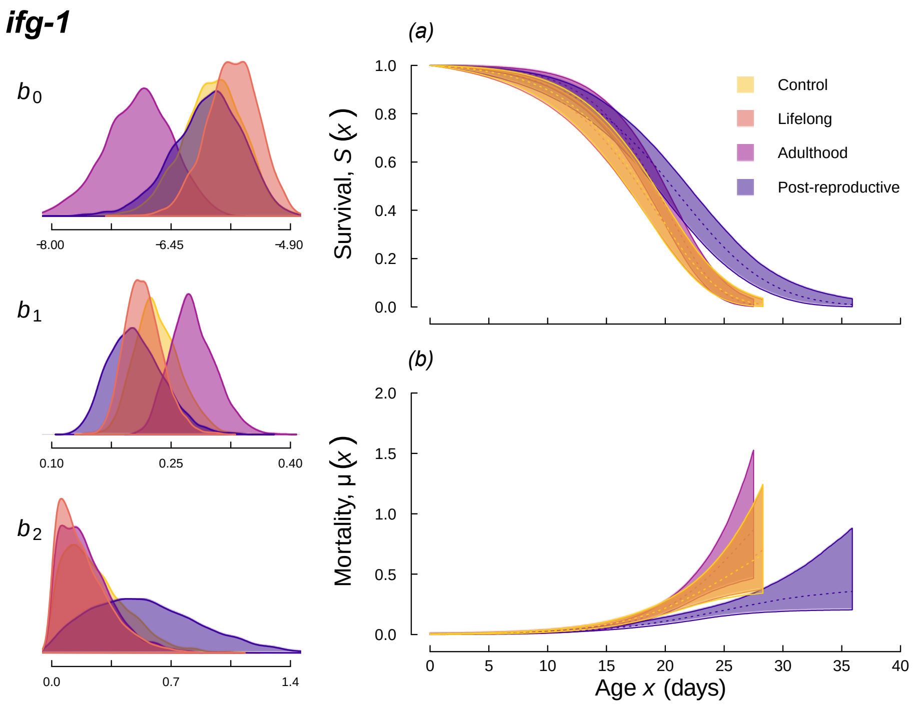

Figure S4. Mortality *ifg-1*. Survival (a) and mortality (b) curves with 95% confidence interval for each treatment, fitted for *ifg-1* using a logistic model with simple shape in *BaSTA*. The left column corresponds to the three mortality parameters; see the method section for a detailed description.

Table S10. Mortality *ifg-1*. Coefficients for the mortality parameters estimated for *ifg-1* using a logistic model with a simple shape.

Coefficient	Treatment	Estimate	SE
b_0	Control	-5.906	0.396
	Adulthood	-6.880	0.442
	Post-reproductive	-5.977	0.453
	Lifelong	-5.653	0.338
b_1	Control	0.233	0.029
	Adulthood	0.278	0.028
	Post-reproductive	0.206	0.034
	Lifelong	0.216	0.024
b_2	Control	0.258	0.186
	Adulthood	0.206	0.154
	Post-reproductive	0.553	0.318
	Lifelong	0.189	0.154

Table S11. Mortality *ifg-1*. KLDC values for each pairwise comparison of every parameter of the mortality rate model for *ifg-1*. Values above 0.8 are considered substantially different and are indicated in bold. See method for details.

Comparison	b_0	b_1	b_2
Adulthood - Control	**0.966**	**0.852**	0.542
Post-reproductive - Control	0.515	0.667	**0.829**
Post-reproductive - Adulthood	**0.934**	**0.962**	**0.901**
Lifelong - Control	0.616	0.612	0.559
Lifelong - Adulthood	**0.995**	**0.969**	0.502
Lifelong - Post-reproductive	0.676	0.587	**0.909**

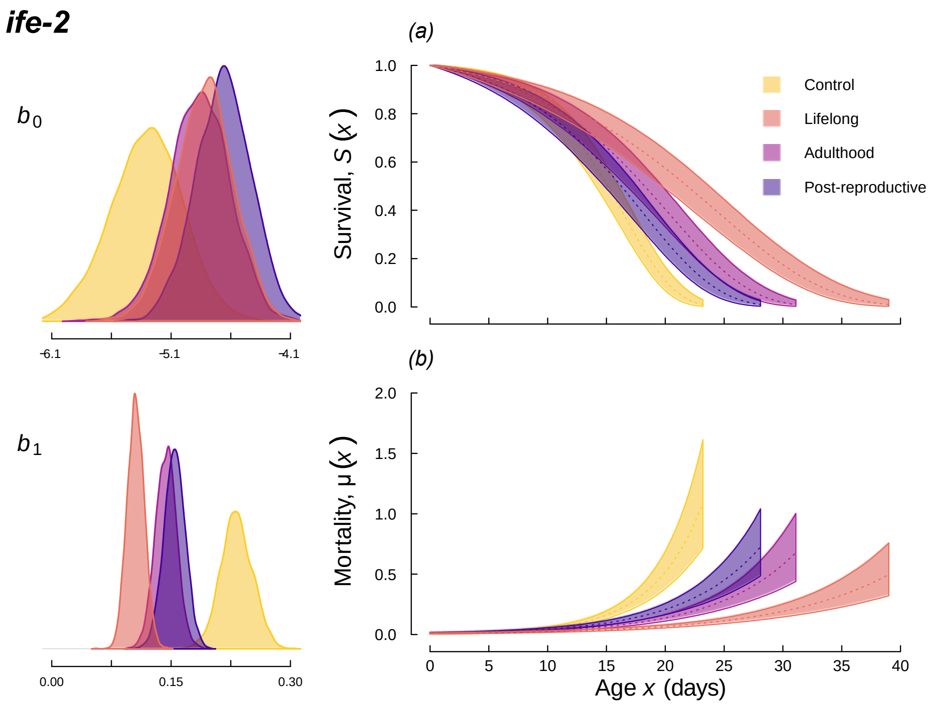

Figure S5. Mortality *ife-2*. Survival (a) and mortality (b) curves with 95% confidence interval for each treatment, fitted for *ife-2* using a Gompertz model with simple shape in *BaSTA*. The left column corresponds to the two mortality parameters; see the method section for a detailed description.

Table S12. Mortality *ife-2*. Coefficients for the mortality parameters estimated for *ife-2* using a Gompertz model with a simple shape.

**Coefficient**	**Treatment**	**Estimate**	**SE**
b_0	Control	-5.306	0.301
	Adulthood	-4.859	0.252
	Post-reproductive	-4.660	0.235
	Lifelong	-4.822	0.245
b_1	Control	0.232	0.020
	Adulthood	0.144	0.013
	Post-reproductive	0.154	0.013
	Lifelong	0.106	0.011

Table S13. Mortality *ife-2*. KLDC values for each pairwise comparison of every parameter of the mortality rate model for *ife-2*. Values above 0.8 are considered substantially different and are indicated in bold. See method for details.

**Comparison**	**b_0**	**b_1**
Adulthood - Control	**0.868**	**1.000**
Post-reproductive - Control	**0.971**	**1.000**
Post-reproductive - Adulthood	0.644	0.634
Lifelong - Control	**0.899**	**1.000**
Lifelong - Adulthood	0.506	**0.995**
Lifelong - Post-reproductive	0.602	**1.000**

Table S14. Fitness. The effect of age-specific down-regulation of each gene on fitness (λ_{ind}). Treatment contrast from mixed-effect models.

Gene	Treatment contrast	coef	SE	d.f.	t	p
age-1	Intercept (Control)	4.318	0.044	83	97.29	**<0.001**
	Lifelong	0.128	0.061	83	2.11	**0.038**
	Adulthood	0.036	0.061	83	0.59	0.556
raga-1	Intercept (Control)	4.342	0.050	2.49	87.52	**<0.001**
	Lifelong	0.033	0.053	86.4	0.62	0.539
	Adulthood	-0.019	0.052	86.3	-0.36	0.717
nuo-6	Intercept (Control)	4.364	0.267	1.68	16.36	**0.008**
	Lifelong	-1.735	0.223	80.0	-7.79	**<0.001**
	Adulthood	-0.396	0.219	80.0	-1.81	0.074
ifg-1	Intercept (Control)	4.148	0.120	78	34.35	**<0.001**
	Lifelong	-4.095	0.176	78	-23.26	**<0.001**
	Adulthood	-1.880	0.167	78	-11.29	**<0.001**
ife-2	Intercept (Control)	4.339	0.056	2.0	76.90	**<0.001**
	Lifelong	0.045	0.054	85.3	0.83	0.411
	Adulthood	0.125	0.054	85.1	2.32	**0.023**

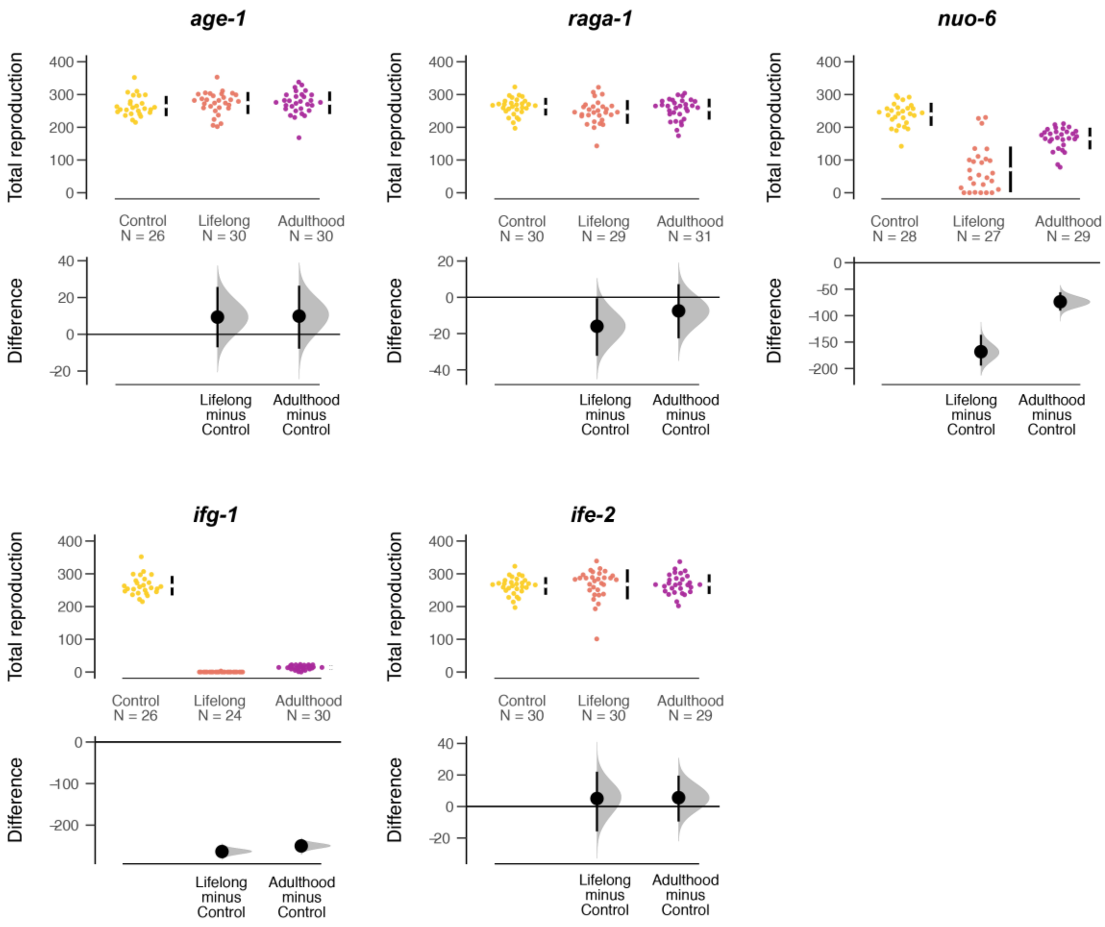

Figure S6. Age-specific effect of “longevity” genes in lifetime reproduction. Lifetime reproductive success (LRS) separated by gene and treatment group: control (yellow), lifelong RNAi treatment (orange) and RNAi during adulthood only (purple). Top panels show raw data, with the mean ± 95%CI indicated by black bars at each group. Bottom panels show estimation plots, where RNAi treatments are compared to the control, with a graded sampling distribution of bootstrapped values and the bootstrapped 95% CI.

Table S15. LRS. The effect of age-specific down-regulation of each gene on lifetime reproductive success. Treatment contrast from generalized mixed-effect models with a Conway-Maxwell-Poisson (CMP) distribution (models with CMP distribution had lowest AIC for all genes, see Table S7).

Gene	Treatment contrast	coef	SE	d.f. resid.	z	p
age-1	Intercept (Control)	5.580	0.033	81	167.85	**<0.001**
	Lifelong	0.032	0.031	81	1.04	0.299
	Adulthood	0.034	0.031	81	1.10	0.271
raga-1	Intercept (Control)	5.556	0.039	85	141.99	**<0.001**
	Lifelong	-0.053	0.031	85	-1.73	0.083
	Adulthood	-0.022	0.030	85	-0.73	0.468
nuo-6	Intercept (Control)	5.482	0.082	79	67.28	**<0.001**
	Lifelong	-1.219	0.126	79	-9.66	**<0.001**
	Adulthood	-0.373	0.098	79	-3.82	**<0.001**
ifg-1	Intercept (Control)	5.576	0.031	75	181.62	**<0.001**
	Lifelong	-7.650	0.599	75	-12.76	**<0.001**
	Adulthood	-2.959	0.089	75	-33.41	**<0.001**
ife-2	Intercept (Control)	5.556	0.042	84	133.75	**<0.001**
	Lifelong	0.028	0.033	84	0.84	0.403
	Adulthood	0.016	0.033	84	0.48	0.628

Table S16. Egg size. The effect of age-specific down-regulation of each gene on egg size. Treatment contrast from mixed-effect models.

Gene	Treatment contrast	coef	SE	d.f..	t	p
age-1	Intercept (Control)	1.26 e-3	1.07e-4	1	11.84	0.052
	Lifelong	-3.81e-6	1.73e-5	68.5	-0.22	0.826
	Adulthood	-8.36e-6	1.64e-5	69.6	-0.51	0.612
raga-1	Intercept (Control)	1.09e-3	2.66e-5	1.40	41.18	**0.004**
	Lifelong	3.39e-5	1.88e-5	27.1	1.80	0.083
	Adulthood	1.18e-5	1.88e-5	27.0	0.63	0.535
nuo-6	Intercept (Control)	1.41e-3	6.43e-5	1	21.89	**0.022**
	Lifelong	6.11e-5	2.45e-5	26.2	2.50	**0.019**
	Adulthood	-7.27e-5	2.44e-5	25.8	-0.30	0.768
ifg-1	Intercept (Control)	1.26e-3	1.09e-4	1	11.58	0.054
	Lifelong	*No eggs produced*				
	Adulthood	1.95e-4	1.59e-5	50.3	12.31	**<0.001**
ife-2	Intercept (Control)	1.10e-3	3.40e-5	1.17	32.17	**<0.001**
	Lifelong	1.68e-5	1.62e-5	30	1.03	0.309
	Adulthood	4.00e-5	1.59e-5	30	2.52	**0.017**

Table S17. Comparisons of models of LRS with different error distribution (Poisson, Conway-Maxwell-Poisson [CMP]), as well as a Poisson model with subject level random effects. If significant zero-inflation was detected, it was modelled using zero-inflated CMP (ZICMP) models. The model with the lowest AIC was selected. Model results are presented in table 1.

Gene	Distribution	Zero infl. model	Disp. ratio	Disp. test, p-value	Zero infl. ratio	Zero infl. Test, p-value	d.f.	dAICc
age-1	Poisson		1.83	<0.001	NA	1.000	4	111.1
	Poisson + obs. level effect		1.01	0.784	NA	1.000	5	2.0
	**CMP**		**1.01**	**0.824**	**NA**	**1.000**	**5**	**0.0**
raga-1	Poisson		1.77	0.016	NA	1.000	4	105.5
	Poisson + obs. level effect		1.03	0.704	NA	1.000	5	4.0
	**CMP**		**1.02**	**0.768**	**NA**	**1.000**	**5**	**0.0**
nuo-6	Poisson		3.00	<0.001	infinity	<0.001	4	1811.2
	Poisson + obs. level effect		0.21	<0.001	312.50	<0.001	5	136.0
	CMP		0.74	<0.001	138.89	<0.001	5	38.5
	ZICMP	~1	0.74	<0.001	1.02	1.000	6	7.2
	**ZICMP**	**~Treatment**	**0.84**	**0.032**	**1.01**	**1.000**	**8**	**0.0**
ifg-1	Poisson		1.57	0.008	1.13	0.112	4	57.8
	Poisson + obs. level effect		0.87	0.384	1.13	0.072	5	35.5
	CMP		0.95	0.792	1.13	0.128	5	4.5
	ZICMP	~1	0.73	0.72	1.08	0.997	6	4.5
	**ZICMP**	**~Treatment**	**1.04**	**0.728**	**1.00**	**1.000**	**8**	**0.0**
ife-3	Poisson		1.89	<0.001	NA	1.000	4	161.7
	Poisson + obs. level effect		0.96	0.8	NA	1.000	5	12.8
	**CMP**		**1.00**	**0.976**	**NA**	**1.000**	**5**	**0.0**

Table S18. Comparisons of models of Age-specific reproduction with different error distribution (Poisson, Conway-Maxwell-Poisson [CMP]), as well as a Poisson model with observation-level random effects. We tested for zero-inflation and over/under-dispersion using the *DHARMa* package. If significant zero-inflation was detected, it was modelled using zero-inflated CMP models (ZICMP). If significant dispersion was detected (if necessary even after modelling zero-inflation), we further included CMP models with different dispersion models, where dispersion was allowed to vary with the level of the covariate (Age and Age^2).The model with the lowest AIC was selected.

Gene	Distribution	Zero-infl. model	Disp. model	Disp. ratio	Disp. test, p-value	Zero infl. ratio	Zero infl. Test, p-value	d.f.	dAICc
age-1	Poisson			1.47	< 0.001	NA	1.000	11	1111.4
	Poisson + obs. level effect			0.53	< 0.001	NA	1.000	12	203.8
	CMP			0.83	< 0.001	NA	1.000	12	115
	CMP		Age	0.87	0.008	NA	1.000	13	68.3
	**CMP**		**Age + Age^2**	**0.96**	**0.512**	NA	1.000	**14**	**0**
raga-1	Poisson			1.47	< 0.001	NA	1.000	11	1141.2
	Poisson + obs. level effect			0.49	< 0.001	NA	1.000	12	200.3
	CMP			0.83	< 0.001	NA	1.000	12	77.7
	CMP		Age	0.88	0.008	NA	1.000	13	21
	**CMP**		**Age + Age^2**	**0.97**	**0.544**	NA	1.000	**14**	**0**
nuo-6	Poisson			0.24	< 0.001	10.32	< 0.001	11	674.7
	Poisson + obs. level effect			0.23	< 0.001	7.73	< 0.001	12	124.5
	CMP			0.47	< 0.001	5.49	< 0.001	12	72.8
	CMP		Age	0.44	< 0.001	4.45	< 0.001	13	55.4
	CMP		Age + Age^2	0.44	< 0.001	4.88	< 0.001	14	57.4
	ZICMP	~1		0.73	< 0.001	1.19	0.472	13	65.3

	ZICMP	~Treatment		0.83	< 0.001	1.00	1.000	15	30.7
	ZICMP	~Treatment	Age	0.83	0.016	1.00	1.000	16	2.4
	**ZICMP**	**~Treatment**	**Age + Age^2**	**0.81**	**0.016**	**0.98**	**1.000**	**17**	**0**
ifg-1	*Almost complete cross-separation, not possible to model*								
ife-2	Poisson			1.22	0.048	NA	1.000	11	979.3
	Poisson + obs. level effect			0.45	< 0.001	NA	1.000	12	234.5
	CMP			0.79	< 0.001	NA	1.000	12	100.6
	CMP		Age	0.86	0.008	NA	1.000	13	29.1
	**CMP**		**Age + Age^2**	**0.96**	**0.576**	NA	1.000	**14**	**0**

Table S19. Primer sequences. Forward (fwd) and reverse (rev) sequences are listed in the 5’ to 3’ direction. Sequences acquired from (Chauve et al. 2020) for target genes and (Akay et al. 2017) for the *actin-3* reference gene.

Gene	Primer Sequences
age-1	Fwd: CGACGTATCTCGCAGATGCA<br>Rev: TTGCCATTCTCGGTCTCCAG
raga-1	Fwd: CCAACAATCGAAGTTGAGCAT<br>Rev: AATGATTCCTGACCACCACAA
nuo-6	Fwd: ATGAATACAATCTGAGCGACGA<br>Rev: CGACGGAGATATTCCTTCTTCA
ifg-1	Fwd: ATCACTATTATCCGCCACAAGC<br>Rev: GGTAGCCTTGGTACTGTTGATTG
ife-2	Fwd: ACGACTCTATTAAGCCGCCAA<br>Rev: GCCATCTTCCTCCATTCTGA
actin-3	Fwd: CCAAGAGAGGTATCCTTACCCTCAA<br>Rev: AAGCTCATTGTAGAAGGTGTGATGC

Table S20. Shapiro-Wilk’s normality test for ΔCt. The ΔCt value (expression of the target gene relative to a reference gene, *actin-3*) was normally distributed for all genes ($p > 0.05$) enabling parametric analysis. Although there was indication of non-normality for *ife-2* ΔCt data separately, visual inspection of the quantile-quantile plot satisfied the normality assumption.

Gene	W	p
all genes combined	0.984	0.912
age-1	0.886	0.296
raga-1	0.960	0.816
nuo-6	0.909	0.432
ifg-1	0.939	0.653
ife-2	0.770	0.031

Table S21. Relative gene expression (ΔCt). The effect of RNAi down-regulation of each gene from the egg stage on expression of the gene of interest relative to the *actin-3* reference gene (ΔCt from qRT-PCR) at day 2 of adulthood. Treatment and gene contrast from linear model analysis of variance table.

Factor	Sum Sq	Mean Sq	F	d.f.	p
RNAi treatment	3.319	3.319	12.186	1	**0.002**
Gene	32.982	8.245	30.277	4	**<0.001**
RNAi treatment x Gene	0.832	0.208	0.764	4	0.561

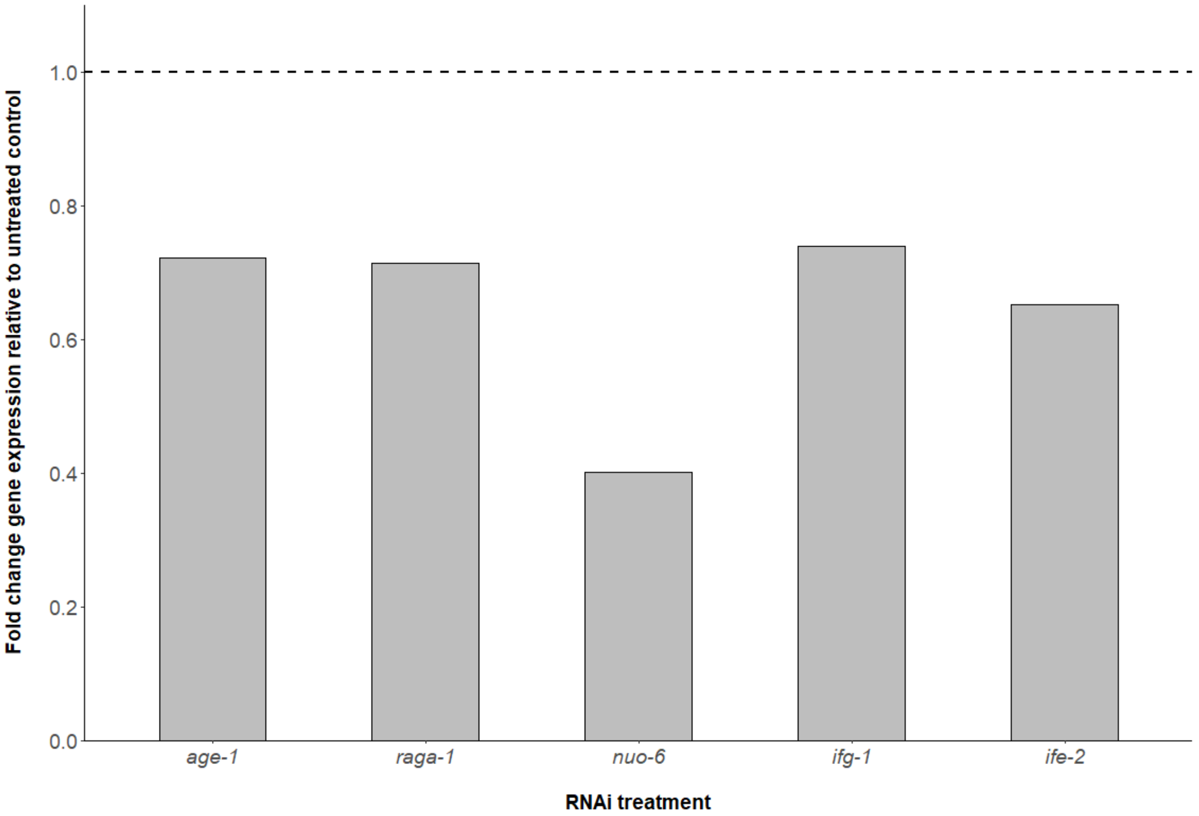

Figure S7. Fold change in gene expression after RNAi treatment relative to untreated controls. RNAi was delivered from the egg stage and gene expression was quantified in two day old adults using qRT-PCR, in three biological replicate pools of 30 worms per RNAi treatment. Fold change calculated as $2^{-\Delta\Delta CT}$ using comparative Ct method (Schmittgen and Livak 2008), from mean ΔCT values for each RNAi treatment. The dotted line at a fold change of one, indicates the expression of the five genes in worms unexposed to RNAi knockdown.

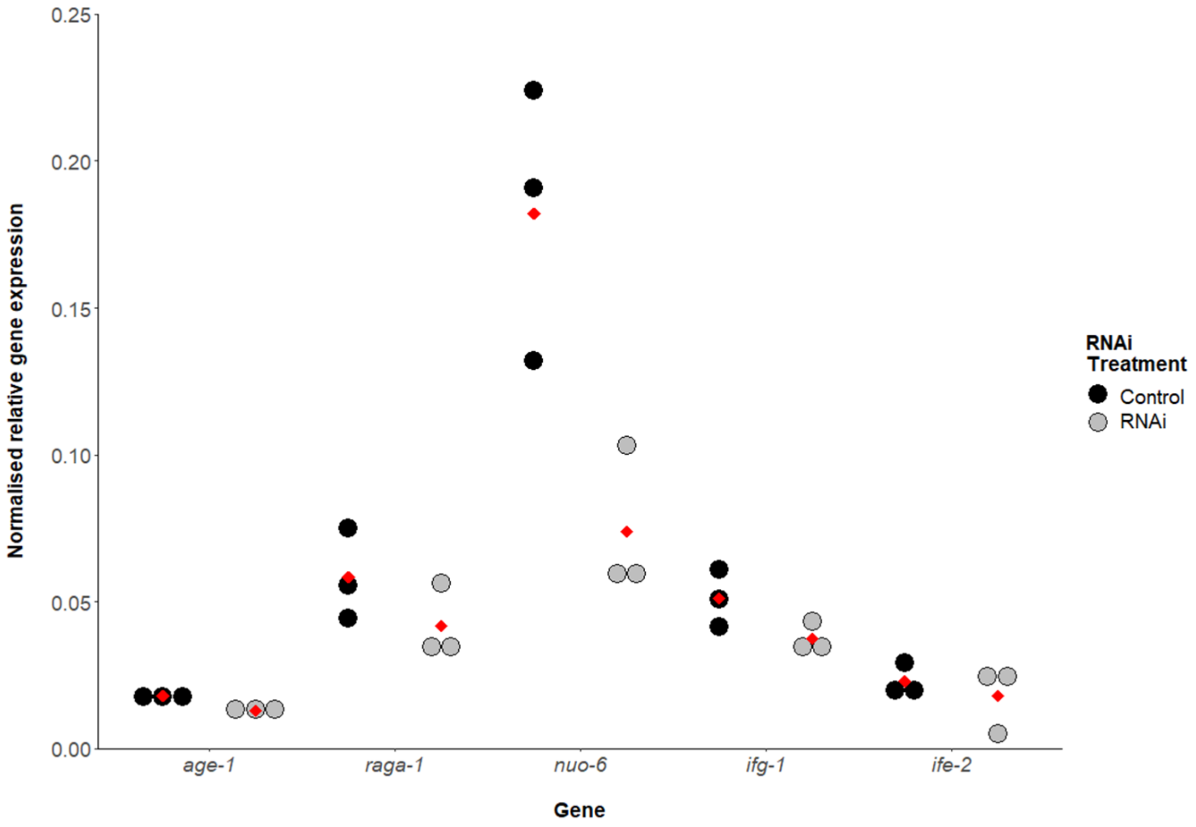

Figure S8. Normalised target gene expression following RNAi treatment versus untreated controls. RNAi was delivered from the egg stage and gene expression was quantified in two day old adults using qRT-PCR, in three biological replicate pools of 30 worms per RNAi treatment (separate points). Arithmetic mean of biological replicates shown as a red diamond. Normalised target gene expression ($2^{-\Delta CT}$) was calculated relative to expression of the *actin-3* reference gene (as Schmittgen and Livak 2008).

References

Akay, A., T. D. Domenico, K. M. Suen, A. Nabih, G. E. Parada, M. Larance, R. Medhi, A. C. Berkyurek, X. Zhang, C. J. Wedeles, K. L. M. Rudolph, J. Engelhardt, M. Hemberg, P. Ma, A. I. Lamond, J. M. Claycomb, and E. A. Miska. 2017. The helicase Aquarius/EMB-4 is required to overcome intronic barriers to allow nuclear RNAi pathways to heritably silence transcription. Dev. Cell 42:241-255.e6.

Bustin SA, Benes V, Garson JA, Hellemans J, Huggett J, Kubista M, Mueller R, Nolan T, Pfaffl MW, Shipley GL, Vandesompele J, Wittwer CT. The MIQE guidelines: minimum information for publication of quantitative real-time PCR experiments. Clin Chem. 2009 Apr;55(4):611-22. doi: 10.1373/clinchem.2008.112797. Epub 2009 Feb 26. PMID: 19246619.

Chauve, L., J. L. Pen, F. Hodge, P. Todtenhaupt, L. Biggins, E. A. Miska, S. Andrews, and O. Casanueva. 2020. High-Throughput Quantitative RT-PCR in Single and Bulk C. elegans Samples Using Nanofluidic

Technology. JoVE J. Vis. Exp. e61132.

Schmittgen, T. D., and K. J. Livak. 2008. Analyzing real-time PCR data by the comparative C T method. Nat. Protoc. 3:1101–1108.

Senchuk MM, Dues DJ, Schaar CE, Johnson BK, Madaj ZB, Bowman MJ, et al. (2018) Activation of DAF-16/FOXO by reactive oxygen species contributes to longevity in long-lived mitochondrial mutants in *Caenorhabditis elegans*. PLoS Genet 14(3): e1007268. https://doi.org/10.1371/journal.pgen.1007268

Weick EM, Sarkies P, Silva N, et al. PRDE-1 is a nuclear factor essential for the biogenesis of Ruby motif-dependent piRNAs in C. elegans. *Genes Dev*. 2014;28(7):783-796. doi:10.1101/gad.238105.114
